# It's Complicated—Adolescent Grief in the Time of Covid-19

**DOI:** 10.3389/fpsyt.2021.638940

**Published:** 2021-02-23

**Authors:** Louis Weinstock, Dunja Dunda, Hannah Harrington, Hannah Nelson

**Affiliations:** ^1^Apart of Me, London, United Kingdom; ^2^Psychology Department, University of Loughborough, Loughborough, United Kingdom

**Keywords:** adolescent, complicated grief (CG), disadvantage, digital, community

## Abstract

Presently, there is a real possibility of a second pandemic occurring: a grief pandemic. There are estimated to be over 1 million children and young people experiencing bereavement because of Covid-19. Adolescent grief is unique due to bio-psycho-social factors such as increased risk-taking, identity-formation, and limited capacity for emotional regulation. In this article, we will argue that adolescents are at increased risk of developing complicated grief during the Covid-19 pandemic, and that it is vital that services are improved to recognize and address this need before secondary problems emerge, including anxiety, depression, and substance abuse. Complicated grief in adolescents is widely underrecognized and often misdiagnosed as a range of mental health problems, addictions, and offending behavior. For example, 25% of <20 year olds who commit suicide have experienced childhood bereavement, whilst 41% of youth offenders have experienced childhood bereavement; this is in comparison with only 4% of the general population. Many of the broader risk factors for complicated grief were already increasing prior to the Covid-19 pandemic, including increased loneliness amongst young people, and the collapse of collective structures to help people manage grief. We propose that this pandemic could be a catalyst for mental health professionals to support and nurture the caring communities emerging in this time as an essential resource to prevent the onset of a grief pandemic.

## Introduction

Currently, Covid-19 has resulted in over 2 million deaths globally (1). Whilst attention has mainly focused on overcoming the virus, there is an urgent need to prepare for the prospect of a “grief pandemic” (2). It is estimated that for every Covid-19 death, 2 children and 4 grandchildren are bereaved (2). Whilst people often show resilience in the face of loss (3), we believe that adolescents—especially those from disadvantaged backgrounds—are at increased risk of developing complicated grief (CG) during this pandemic. A literature review on the impact of loss on disadvantaged groups concluded that “death never occurs in a vacuum but within a social context, the nature of which can influence greatly how the person deals with that loss” (4). This article aims to highlight the current global context that is increasing the risk of CG in adolescents, alongside strategies to reduce this risk.

## What is Grief Anyway?

Grief is a form of emotional energy that human beings experience following the death of something or someone. Grief is found in all human cultures, and a grief-like response has been identified in some animals too (5). Grief seems to be an emotion for social creatures, particularly those with a limbic system—the area of the brain primarily responsible for emotion and memory (6). For humans, there is a significant opioid response correlated to a heightened social connection with loved ones; some neuroscientists declared that such connections were “... in some fundamental neurochemical sense, opioid addictions” (7). In contrast, when social contact is severed, creatures with a limbic system experience suffering akin to opioid withdrawal. Typically, humans will react by trying to re-establish or repair the contact. This is especially true for the young, and when reconnection is not viable, as with death (8).

How we deal with grief varies between individuals and cultures. In the Orthodox Jewish tradition, sexual activity is forbidden during the mourning period, yet the Cubeo tribe of the northern Amazon include sexual activity as part of the wake (9, 10). In urban Senegal, it is common for the bereaved to be criticized if they display excessive emotion (11). In addition, individual factors such as attachment style, family systems, and type of death, all shape the grieving process (12, 13). A combination of these factors means that an individual's lived experience of loss will suffocate if held too tightly in any universal psychology of grief.

**Figure 1:**
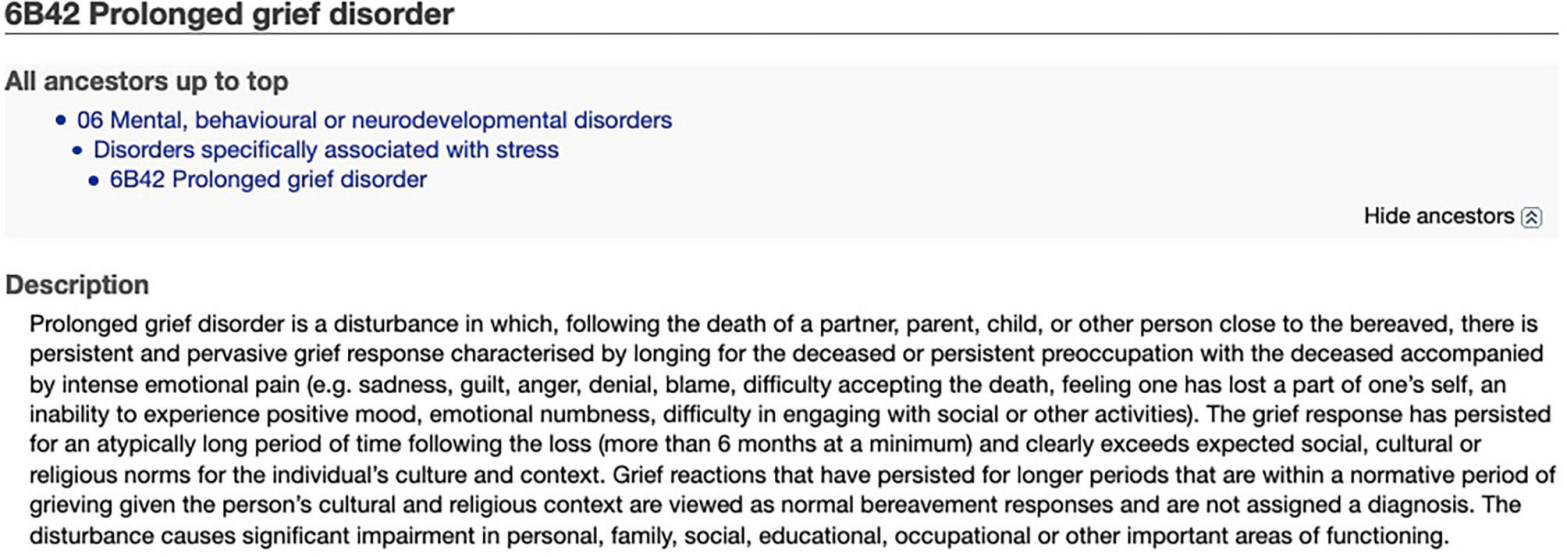
Diagnostic criteria for Prolonged Grief Disorder from the ICD-11 (16).

The first diagnostic criteria for a bereavement-related disorder were introduced in 1993 by the psychiatrist Mardi Horowitz. CG described patients who were experiencing intrusive thoughts about the deceased, avoidance, and intense negative emotions (14). Several factors were identified that increased the risk of CG, including social isolation, increased anxiety, and experiencing a sudden and inexplicable loss (15). The Covid-19 pandemic has heightened many of these risk factors. Since the original definition of CG, the ICD-11 has adopted a diagnosis of “Prolonged Grief Disorder” (Figure 1).

## Adolescence in Context

Adolescence is a unique, highly sensitive developmental stage initiated by puberty. During this time, the biological imperative is to detach from family and move closer to peers. Adolescence is also a particularly high-risk stage for the onset of mental health problems (17). Many cultures developed rites of passage to support this transition, although these are less common in the modern world. Many young people have created their own customs, such as gang initiations (18–20). In more individualistic cultures, a core developmental task of adolescence is to determine “who am I?” Adolescents seek a coherent, integrated, and stable sense of themselves, separate from the identity imposed on them by family. This quest is complicated in the 21st Century, as the range of possible identities continuously expands.

If a significant loss disrupts these developmental tasks, the transition to adulthood becomes complicated (21). Keenan argued that bereaved young people deal with a “double dose” of obstacles: adolescent challenges and bereavement challenges (22). Moreover, research increasingly suggests that adolescents grieve differently to adults, and that adolescent grief is commonly misunderstood (23, 24).

Grief during adolescence can be viewed as an unanticipated rite of passage, an initiation that disrupts the quest for identity and “requires us to die to the old image of who we thought we were and step across the threshold into a radically altered sense of self” (25). Without a solid support system, the initiation of adolescent grief can become troublesome. Adolescents who have experienced loss are more likely to experience difficulties in the work-place and have diminished educational achievements and aspirations (26). They are also at increased risk of psychological disorders (27), particularly depression (28), and physical illness (29). Evidently, loss can seriously hinder quality of life for adolescents.

Adolescents exhibit a distinct pattern of neurological development. Combining the neurological understanding of both grief and adolescence can help identify why grief is problematic during this stage and can establish why adolescents are prone to develop CG. Pain-related and reward-related pathways both contribute to the grieving process (31, 32). As social connections have been compared to opioid addictions, O'Connor et al. conceptualized CG as an addiction. Memories of the deceased activate reward-related pathways, thereby prolonging the grieving process. A recent study found that when patients with CG saw photos of their deceased loved ones, the nucleus accumbens—associated with rewards or longing—was stimulated significantly more than people who experienced “normal” grieving. O'Connor said: “It's as if the brain were saying, “Yes I'm anticipating seeing this person” and yet “I am not getting to see this person.The mismatch is very painful” (32). Adolescents experience high levels of sensation-seeking and activation of reward-pathways, and consequently, they are more vulnerable to risk-taking behaviors (30) and developing addictions (33, 34). Neurologically, adolescents appear more vulnerable to developing CG.

## The Context of Grief Pre-Covid

There are four core cultural shifts that were existent pre-Covid and were already significantly impacting the grieving process of adolescents: (1) the stigmatization of grief, (2) the fragmentation of traditional community structures and the rise of individualism, (3) the loneliness epidemic, and (4) the proliferation of digital technologies.

### The Stigmatization of Grief

Socio-cultural norms around grief influence the grieving process. Over the last two centuries, the average human life-span has increased rapidly, and expectations around death and grief have changed correspondingly (35, 36). In Victorian Britain, grievers would view the body after death. Today, it is normal to have a closed casket at a funeral (35). Additionally, images of mourning proliferated in Victorian Britain; by the 1870s in the USA, girls could order dolls complete with coffins and mourning clothes (36). Traditionally in Judaism, the loss of a parent results in a year-long grieving process. Yet in the 21st Century, many establishments in the UK and the USA fail to provide any bereavement policies. The average length of compassionate leave in the UK is 2–5 days, but 40% of workers do not receive time off to handle family bereavement (37, 38). It seems the main message around grief in Advanced Capitalist societies is to hurry and get on with it. Research suggests that the new diagnosis of prolonged grief disorder can lead to greater stigmatization. In one study, participants reported increased anger and anxiety, and desired greater social distance when this diagnosis was mentioned (39). One thing grieving adolescents do not need at this time is increased social distance.

### Community Fragmentation and Individualism

Over thousands of years and across many cultures, collective structures have evolved to guide humans through grief (40). Yet, in modern, secular societies these structures have been fragmented, leaving populations severed from the language and practices that historically guided them through loss. Individualism is now on the rise globally and is likely a contributor to this structural fragmentation (41). In individualistic cultures, grief is viewed as an intrapsychic phenomenon, as opposed to social or cultural (42). As humans are social creatures, and grief is a social emotion, this trend is problematic. Some researchers suggest that grief counseling is merely a continuation of this trend, another way of sequestering grief into private spaces. Walter proposed that: “Counseling and self-help groups collude with the private modern way of death, keeping grief conveniently out of the everyday way so that life can go on as though death did not exist” (43).

### Loneliness Epidemic

In the UK, USA, and parts of Europe, young people feel more alone than any other age groups (44, 45). There is evidence of a positive correlation between the individualistic nature of society and loneliness (46). For young people in modern cultures, grief can be a lonely experience, particularly as conversations about death are often discouraged (47). Loneliness can have devastating consequences; one recent study found that “people who report feeling lonely after a sudden bereavement are more likely to make a suicide attempt after their loss” (48). This sits uncomfortably with the finding that, in the UK, 25% of <20 years old who commit suicide had experienced a childhood bereavement (49).

### Proliferation of Digital Technologies

Half of the global population have regular access to a smartphone, especially young people (50, 51). The amount of time spent online is also growing. Over a third of 15-year-olds in the UK are “extreme internet users,” online for at least 6 h a day at weekends; in the USA, 95% of teenagers have access to a smartphone, and 45% are online “almost constantly” (52, 53).

Creative examples are emerging of young people expressing their grief online, from memorials in Minecraft to expressions of collective grief on social media, seen most poignantly in the aftermath of the George Floyd killing (54). Social media offers many advantages for grieving adolescents: there is little friction—a young person can announce their grief with a simple status update and instantly receive supportive responses; it is a space they are familiar with; for those who feel marginalized in everyday life, online communities allow them to feel empowered in their grief; and finally, social media provides a platform for creative expressions of grief (55, 56).

However, the expression of grief online also presents limitations. Grief needs to be managed in a safe environment, which is not guaranteed online: a single social media post can create an emotional storm, sending shock-waves throughout our nervous systems (57). The developing brains and nervous systems of young people are constantly exposed to the harsh realities of the world through social media, including death and potential civilizational collapse. It is likely they are significantly affected by this emotional contagion (58).

Individuals with CG exhibit increased neural activity of reward-related pathways. Numerous studies have demonstrated how social media and other digital platforms are designed to attract us by eliciting our dopamine-fueled, reward-seeking neural pathways (59, 60). Therefore, young people likely find it harder to grieve in this digital age.

When a loved one dies, we have access to photos, videos, and messages that maintain our personal connection with them. However, this can prolong the process of letting go. “Ambiguous loss” describes loss without closure or understanding. In this digital age, the ongoing existence of the deceased's virtual self makes it harder to obtain closure (61).

## Why is Covid-19 Making Grief More Complicated for Adolescents?

Whilst we cannot confirm the long-term mental health effects of Covid-19 on grieving adolescents, this pandemic has accelerated many of the described risk factors for CG. Social isolation has now become a global policy in the form of social distancing and physical lockdowns, raising concerns about the impact on young people's mental health (62, 63). Collective structures for grieving have been difficult or impossible to access (64). Young people have been forced to say goodbye to loved ones via iPads and have been denied the basic comfort of a hug from anyone outside their immediate family (65).

As young people have spent more time distanced from their peers, one might predict social media usage would skyrocket. However, the reality is complicated. A study of over 1,500 teenagers in the USA showed that social media consumption decreased overall during this pandemic. Furthermore, the teenagers who received more sleep and more family time due to the lockdown measures reported better mental health than pre-Covid (66).

Nonetheless, the same study showed that teenagers whose parents had become unemployed, had financial worries, or were concerned about food supplies, reported far higher levels of depression. The increased mental health burden of Covid-19 on disadvantaged youth is echoed in other studies (67). Pre-Covid, research had established that young people from disadvantaged backgrounds were more likely to lose a parent early in life, and were more likely to suffer from CG due to fewer opportunities “to process immediate difficulties before the next adversity strikes” (4, 68, 69). Covid-19 has impacted disadvantaged communities significantly, regarding morbidity and the economy. Consequently, a significantly elevated risk of CG for young people from disadvantaged backgrounds is expected (70), unless the mental health community can reverse this trajectory.

Given that CG and related grief disorders can only be diagnosed at least 6 months after a loss, it may be too early for data to support our hypothesis. The pressure on the NHS for death prevention may have also distracted from diagnoses. Although we currently do not know how Covid-19 will impact CG levels, given the traumatic and unexpected nature of Covid-19 deaths, it has been predicted that these values will rise (71).

## Hope

Adolescent grief during Covid-19 is certainly complicated, but hope is a critical resource that can turn a traumatic event into an opportunity for post-traumatic growth (72). So, in these most challenging of times, how can we find and nurture hope for bereaved adolescents? Here, we consider two pathways:

### Clinical

CG treatments have been developed based on cognitive behavior therapy and interpersonal psychotherapy (73). These therapies aim to free the normal grieving process, rather than free the individual of grief (73). Intervention therapies are promising; a meta-analysis of 14 studies concluded that intervention treatments yielded significantly positive results (74). Contrarily, preventative measures for CG are currently ineffective. Further research into the prevention of CG is essential, focusing on risk and protective factors for which there is good evidence (74).

However, managing these factors in a pandemic requires the adaptation of services, such as offering more services digitally (75). Whilst the ability of digital services to adequately address the risk factors for CG is unknown, it is believed that isolation/loneliness primarily requires face-to-face support. Currently, there is insufficient research to empirically assess the limitations of tele-psychiatry (76).

Encouragingly, the pandemic may alleviate some risk factors, especially in families with positive relationships. A strong relationship with a surviving parent is a protective factor for CG (77); lockdown may have contributed to improving existing parent-child relationships (66). Improved awareness of the risk factors and potential for CG among healthcare professionals will be key to helping those affected both during and after the pandemic.

### Social

We have shown that many of the social factors increasing the risk of CG in this pandemic were already increasing pre-Covid-19. This pandemic has exposed the mental health burden that arises in societies where grief is stigmatized, social isolation and digital immersion are pervasive, and collective structures for healing and mutual support are withering.

Just as grief can be seen as an unanticipated initiation for adolescents, this pandemic can be viewed as a collective initiation. Indeed, COVID-19 has been described as “an unprecedented catalyst for social transformation” (78). By revealing these implicit social structures and our shared vulnerabilities, the pandemic has allowed human beings to access deeper levels of compassion—from rainbows in windows and clapping for the NHS, to the rise in volunteering and mutual aid groups. Research shows that compassion, empowerment, and deeper connections to others are common responses to mass emergencies (79).

The psychotherapist Darian Leader stated, “mourning requires other people.” A systematic review on loneliness and young people's mental health concluded “finding ways to give children and adolescents a sense of belonging.and to feel that they are part of a wider community should be a priority” (80). This pandemic is providing an opportunity to highlight community structures to help vulnerable young people grieve. We believe that the “Compassionate Communities” model should be studied to support adolescents at risk of developing CG. Compassionate Communities are a growing movement in the UK and Europe, where palliative care providers engage local communities in conversations about death and grief, promoting a literate culture around these topics. In South London, a hospice invited schoolchildren to visit and encouraged them to ask questions like “what happens to your body when you die?” (80).

It is essential that mental health professionals continue supporting this sense of belonging by advocating mutual aid groups and compassionate communities. Research consistently shows that without systemic or structural change, mental health care is inadequate to reverse the detrimental effects of social disadvantages (81, 82). Professional psychoeducational programming, wellness outreach, fundraising for mental health services, and mental health advocacy are all well-documented strategies that can sustain these communities (78).

Furthermore, communities aiming to help young people grieve should have “critical hope” at their core. The pedagogist Paolo Freire distinguished “naive hope” from “critical hope”: a detachment from any sense of agency, empowerment, or political struggle that is likely to disintegrate into hopelessness. “Critical hope” is instead understood as “an action-oriented response to contemporary despair” (83). Initiatives are needed imminently to increase adolescents' critical awareness of social trends that further complicate grief. Such initiatives can alleviate societal pressures to continue as normal, despite facing emotional pain (84). There is increasing evidence for the positive impact of interventions focused on adolescent empowerment and critical consciousness (85–87).

## Conclusion

Grief can be transformed into a powerful catalyst for social change, political action, and collective well-being (88, 89). The Covid-19 pandemic is a powerful opportunity to reinvigorate collective structures that can help vulnerable young people through their darkest moments. This article has highlighted the increased risk of CG for bereaved young people during this pandemic, especially those from disadvantaged backgrounds. Mental health professionals and organizations need to respond to the “grief pandemic” by nurturing collective structures that can provide grieving young people with a sense of belonging. Future research should include a literature review on CG and young people, alongside research into the communities that can sustainably generate a vital sense of belonging for young people.

## Data Availability Statement

The original contributions presented in the study are included in the article/supplementary material, further inquiries can be directed to the corresponding author/s.

## Author Contributions

LW, DD, and HH conceptualized and wrote the manuscript. HN contributed to the literature review, and critically reviewed the manuscript. All authors agree to be accountable for the content of the work.

## Conflict of Interest

The authors declare that the research was conducted in the absence of any commercial or financial relationships that could be construed as a potential conflict of interest.
